# Mental health clinicians’ perceptions of nature-based interventions within community mental health services: evidence from Australia

**DOI:** 10.1186/s12913-022-08223-8

**Published:** 2022-06-30

**Authors:** Rachel Tambyah, Katarzyna Olcoń, Julaine Allan, Pete Destry, Thomas Astell-Burt

**Affiliations:** 1 Illawarra Shoalhaven Local Health District, Warrawong, NSW 2505 Australia; 2 The University of Wollongong, Wollongong, NSW 2522 Australia; 3 Illawarra Community Mental Health Rehabilitation Service, Warrawong, NSW 2502 Australia

**Keywords:** Green space, Nature, Nature-based interventions, Mental health, Mental health services, Mental health clinicians

## Abstract

**Background:**

Mental health conditions are one of the largest burdens of disease in Australia and globally. There is a need to seek innovative and alternative interventions that can prevent and alleviate mental health symptoms. Nature-based interventions (NBIs), namely programs and activities where individuals engage with natural environments with the aim of improving their health and wellbeing (e.g., nature walking groups), may be such an alternative. This study aimed to explore the perceptions of mental health clinicians on the potential benefits of, and barriers to, implementing NBIs within a community mental health setting.

**Methods:**

This study used a qualitative, exploratory research design. Fifteen mental health clinicians were recruited from the Illawarra Shoalhaven Local Health District, Australia, and interviewed (September–October 2021) about their perceptions of NBIs within mental health settings. The semi-structured interviews were analysed using an inductive thematic approach and managed with NVivo.

**Results:**

Mental health clinicians viewed spending time in nature as relaxing, refreshing, and therapeutic. Many described it as part of their lifestyle and encouraged consumers to engage in nature-based activities on their own time. If NBIs were to be introduced as part of mental health services, clinicians expressed willingness to promote them to their consumers. Clinicians listed many potential benefits of NBIs for mental health consumers including improved mood, calmness and relaxation, a sense of empowerment, and social connections. Despite these benefits, clinicians were concerned about a variety of barriers to NBIs including consumers’ mental health symptoms such as anxiety or lack of motivation, scepticism, and geographic accessibility, as well as organisational barriers such as policies around safety risk.

**Conclusion:**

Responding to the individual and organisational factors that could hinder the implementation of NBIs while building on the existing evidence of the positive impact of nature on health and wellbeing and, as demonstrated in this study, mental health clinicians’ interest and supportiveness of NBIs, mental health services should consider the implementation of NBIs as part of routine practice.

## Background

Mental health conditions are one of the largest burdens of disease in the world [1]. They impact all aspects of a person’s life, from day-to-day routines involving work and study, to interactions with family and friends [2]. In Australia, mental illness accounts for twelve percent of the total disease burden, and 45% of the country’s population are estimated to experience a mental health condition at some point in their life, with the most common being anxiety, depression, and substance abuse [2]. In 2019–20, Australia spent $11 billion on mental health services [2], despite this high cost, only a third of people with symptoms of a mental disorder accessed mental health services [2]. People with mental illness frequently experience cognitive and functional impairment that is associated with poor health and social outcomes, frequent presentations to hospital, long inpatient admissions, and an ongoing need for support from community-based mental health services [3]. With the rising social and financial burden associated with the prevalence of mental illness, there is an urgent need to explore alternative interventions that can be used alongside, or in some cases as a substitute for, traditional treatments [4, 5]. NBIs can create both personal and economic gain as individuals and health systems can lower the high cost of mental health treatment and the burden placed on mental health professionals evident in long waitlists [6]. Furthermore, the ability to receive mental health support in a natural environment can reframe the clinical approach available to mental health consumers and aid their recovery. Glover’s [7] model of recovery suggests that people with mental health conditions need to be active, participate and take responsibility for their recovery journeys. This recovery focus moves away from the predominant bio-medical model of diagnosis and treatment of mental illness to a contextualised and humanistic view of what constitutes therapeutic intervention.

In the last few years, there has been an increased interest in the health benefits of spending time in nature [8, 9]. Contact with nature and health have been linked in three interconnected domains – reducing harm from exposure to environmental hazards such as noise; restoring capacities diminished by stress and constant stimuli; and building new capacities to interact socially with others and do new things [10]. Nature-Based Interventions (NBIs), namely programs and activities where individuals engage with natural environments with the aim of improving their health and wellbeing, have been increasing as a result [11, 12]. NBIs vary from nature walking groups [13], forest bathing [14], community gardening [15], and talking therapies delivered in a natural setting [16], among others. Their application in mental health settings, however, remains sporadic [17, 18].

### Benefits of nature exposure on mental health

Evidence of the mental health benefits of nature exposure is rapidly growing [19–22]. Contact with nature (e.g., parks) is associated with improvements in memory, cognition, and attention [23, 24], reduction in symptoms of depression and anxiety [25–28], lower stress levels [4, 29–31], and healthy sleep patterns [32]. Studies have also found that NBIs resulted in greater levels of confidence, feelings of self-worth, happiness, feeling of safety, and sense of purpose and empowerment [32–35]. Sempik and Bragg ([36] p101) have claimed that nature’s qualities such as “the views, the smells, the sense of ‘being there’ and sense of place” are therapeutic. They can activate emotional healing by allowing individuals to process their thoughts and emotions nonverbally as they communicate with nature through their senses [37].

Nature exposure has also been linked with lower incidence of loneliness [38–40]. When offered in groups, NBIs created opportunities for social connections alleviating loneliness [27] which is one of the main indicators of mental and social wellbeing [41]. Participating in NBIs with others with similar lived experiences enhanced engagement, contributed to feelings of togetherness and belonging, and improved social skills [27, 35].

Overall, natural environments provide a space for people to rest, recover, and reset [23], and importantly, studies that looked at the longer-term impact of NBIs at six [32] and 12 months [29] after the intervention ended found that well-being outcomes were sustained. Moreover, some research has found evidence of synergies in outcomes, such as various studies reporting the mental health benefits of exercise tend to be higher when participation occurs in nature settings [42, 43].

### The role of mental health clinicians in nature-based interventions

Despite the benefits described above, nature has been underutilised in supporting individuals with mental health conditions [22]. Although the limited knowledge of health practitioners about the benefits of nature has been identified as a barrier to NBIs [12], no studies have been conducted on the views of mental health clinicians on the use of NBIs as part of public community mental health services. Community mental health services in Australia include individual and group counselling, case management, psychiatry and medication review for people with a diagnosed mental illness and are provided by multi-disciplinary teams [2]. The success of NBIs as part of mental health services, whether as a sole intervention or as an adjunctive therapy [44], is largely dependent on mental health professionals’ interest and support as it would be their task to promote and/or implement it. Moreover, the presence and guidance of a qualified mental health practitioner in NBIs, distinguishes it from other types of nature-based programs and activities [36]. Specifically, it ensures that a coordinator “is able to speak (at least) two different ‘languages’: the language of healthcare and practice; and the language of nature and environmental engagement” [25, p 83]. Thus, given that mental health clinicians are instrumental in the development, promotion, and implementation of these NBIs, this study aims to explore their perceptions of the benefits of, supports needed, and barriers to, implementing NBIs within mental health services.

## Methods

### Study setting and design

This study employed qualitative description as articulated by Sandelowski [45, 46], and which is the preferred method of analysis when a comprehensive summary of an event in the terms of those describing it is more important than a researcher driven interpretation. Qualitative description offers interpretive validity, or an accurate accounting of the meanings participants attributed to those events that participants would agree is accurate [45].Semi-structured interviews were conducted with mental health clinicians within Illawarra Shoalhaven Local Health District (ISLHD) to explore their perceptions on implementing NBIs within community mental health services. The term “consumer” is used throughout this article to represent people who use mental health services. This is the preferred term of the New South Wales (NSW) health system where the study was undertaken [47]. All participants provided written consent for participation, and the University of Wollongong Human Research Ethics Committee granted ethical approval. ISLHD spans 250 kms of the southern coastal strip of NSW [48]. It has a population of close to 400,000 residents, and a higher proportion of low-income households compared to the state average [49]. In ISLHD in 2020, 18.4% of adults experienced high or very high levels of psychological distress (compared to 16.7% NSW average), and the death rate from suicide was 17.5 per 100,000 population (11.3 in NSW) [50]. The district provides publicly funded inpatient and community based clinical mental health services [48]. The region, located between the mountains and the sea, is appreciated for its natural beauty, coastal and escarpment areas, the Illawarra Lake, and has many walking trails and National Parks. In ISLHD, 24.6% people were born overseas compared to 34.5% NSW average and the most common birthplaces, other than Australia, were England, New Zealand, Scotland, and Germany [50].

### Recruitment and sample characteristics

The study used purposive sampling to recruit participants who 1) were 18 or older and 2) provided direct mental health services within ISLHD. A Mental Health Executive Officer and Social Work Educator within ISLHD circulated the recruitment letter on behalf of the research team to all ISLHD mental health staff via relevant email lists. Snowball sampling was then utilised where participants would refer the researchers to other potentially interested mental health clinicians. This allowed for recruitment of additional five participants who had not been reached by the initial recruitment strategy.

Fifteen mental health clinicians from ISLHD agreed to be interviewed. Participants had differing roles including social work, nursing, psychology, peer support, and community development. Participants’ age range was 28–61 years (average of 45.7 years), with 80% being female and 80% of various White ethnic backgrounds. Participants had been practicing in mental health settings between 2 and 40 years (average of 15.6 years). The composition of the sample is reflective of the mental health professional community in ISLHD.

### Data collection

Data collection took place in September and October of 2021. All participants took part in semi-structured interviews with the first or the second author that explored their perceptions of the benefits of, and barriers to, implementing NBIs. The interview guide was developed collectively by the whole research team and included open-ended questions such as “To what extent might the mental health consumers you work with benefit from participating in nature walking groups, in the short- and long-term?” and “What do you perceive may be a barrier to utilising nature walking groups for the improvement of mental health?” In addition to asking all questions from the interview guide, the researchers also occasionally utilised prompts, allowing participants to further reflect and expand on their responses. Due to COVID-19 restrictions, the interviews were conducted via Zoom. They ranged from 22 to 66 min in length (average of 38 min). Interviews were recorded and professionally transcribed. The researchers took fieldnotes reflecting on the process and the outcome of each interview, which allowed for monitoring the quality of the interviews and making needed improvements such as rephrasing questions or using alternative prompts [51].

### Data analysis

The data was analysed using an inductive thematic approach [52]. NVivo (QSR International) was used to organise transcriptions and code the data. Inductive thematic analysis involves “starting the analytic process from the data, working ‘bottom up’ to identify meaning without importing ideas” [52 p835]. The first author utilised Braun et al.’s [52] six stage thematic analysis approach which involved familiarisation with the data by reading and re-reading the interview transcripts and utilising NVivo to systematically generate codes. Themes were then constructed, revised, and defined to capture “a meaningful pattern across the dataset” [52 p855] and to gain a strong understanding of how each theme relates to another. 

The development of the final themes was supported and confirmed through the reflective fieldnotes [51]. The credibility of the interpretation of the data also relied on regular peer debriefing [51] with the fourth author who is a mental health clinician affiliated with ISLHD but was not one of the study participants. Regular reviews of the analysis with this author provided the interpretative validity required for the descriptive method [45]. The other authors were all based in the regional university at the time of the data collection and analysis. Specifically, the first author was a social work student who completed this project as part of her honour’s thesis and is currently employed as a social worker in ISLHD. The second and third authors are social work academics who supervised the first author and expanded the data collection and analysis beyond the honours project. The fourth author is a mental health clinician who plans to implement nature-based intervention at his agency and who initiated the research collaboration. The final author is a social and environmental epidemiologist with expertise in the links between green space and health. Any discrepant views among the research team or potential biases were resolved by discussion. Lastly, the researchers strived for transferability by providing rich and detailed descriptions of the research context and thick descriptions of the research findings, supporting readers to assess whether findings are transferable to alternative contexts [53].

## Results

**Fig. 1:**
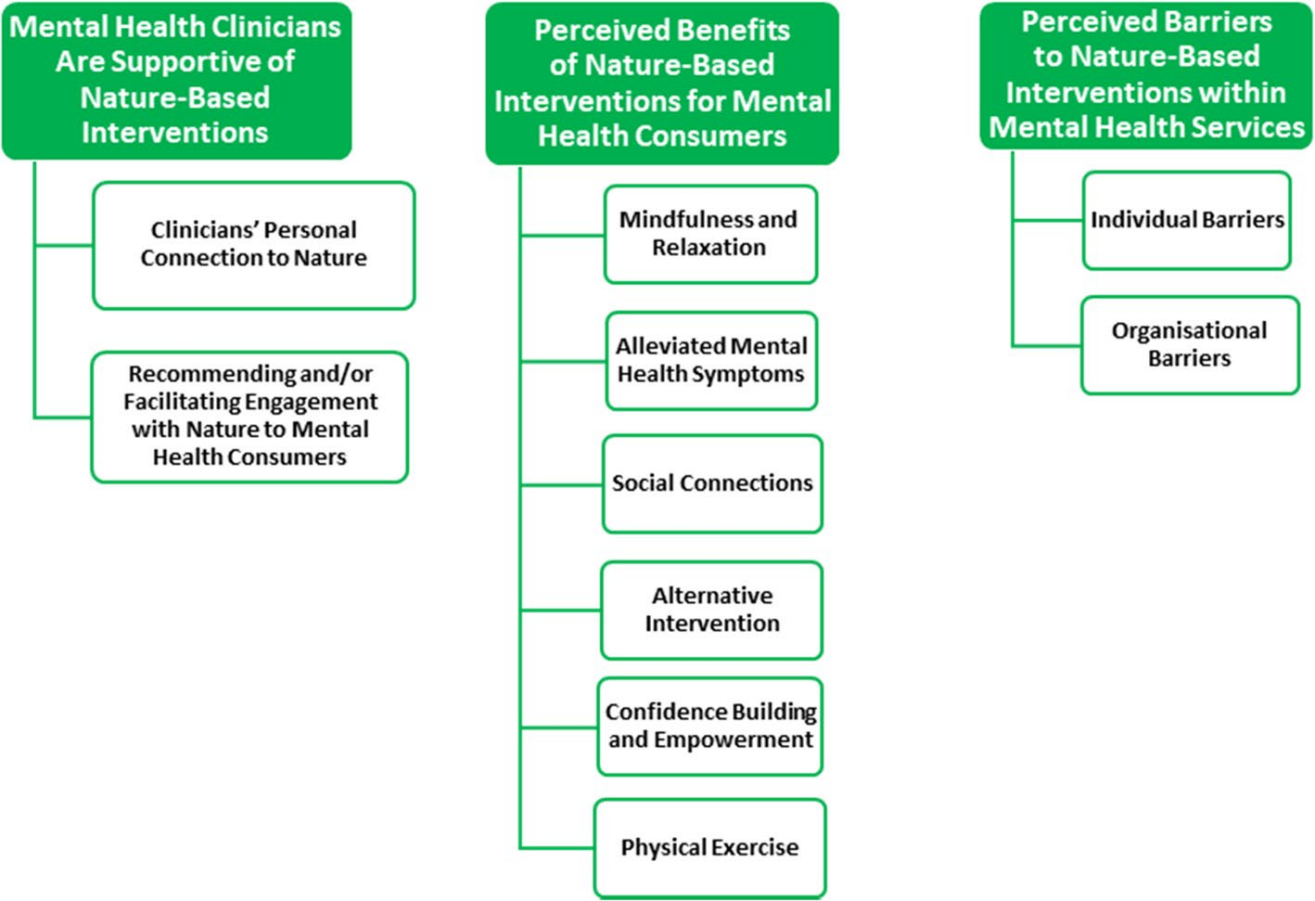
Summary of the Findings

Three major themes were identified in the interviews: 1) mental health clinicians are supportive of NBIs; 2) perceived benefits of NBIs for mental health consumers; and 3) perceived barriers to NBIs within mental health services. To protect the confidentiality of research participants, they will be referred to as “clinicians” in the results and when quoted, they will be identified by their assigned number. Figure 1 summarizes the themes and subthemes that are described in detail below.

### Mental health clinicians are supportive of nature-based interventions

All interviewed mental health clinicians were supportive of NBIs and were willing to recommend these programs to their consumers. When asked about their personal experiences with nature and its potential impacts on mental health, many reflected on their upbringing and how their childhood influenced their love for nature and how it became a part of their lifestyle. They described how they found spending time in nature beneficial for their own health and the therapeutic impact it had for them. Many clinicians were already recommending to their consumers to spend time in nature, and some even facilitated nature exposure as part of their services.

#### Clinicians’ personal connection to nature

Several clinicians described their experiences of growing up surrounded by nature, playing with friends outdoors and exploring and learning about the natural world:

As kids, we lived close to a mountain range. So, we would go bush walking or abseiling or different things like that. And living on a farm, I would always be surrounded by nature or collect wildflowers or different things like that throughout my childhood as well. So, it’s always something that’s been important to me and always been something that’s been part of my life (12).

They had experiences with various natural environments including the bush, costal zones, rivers and lakes, forests, and campsites. The value of time spent in nature growing up became embedded in these clinicians’ lives. Over half of them stated that exercising in nature was part of their weekly routine. Some specifically moved to the Illawarra region because of its proximity to the bush and the beach. They reflected on their experiences of walking in nature, including bushwalks, spending time at the beach or going camping and the impact on their own mental health:

I find [that it] is really supportive for my own wellbeing. It’s something that helps me feel relaxed and recharged and refreshed. Particularly after maybe a stressful period at work…it’s something that I really enjoy and find beneficial for myself (12).

Another clinician reflected on time in nature to allow for a “freeing natural human state of just being” (11) that they actively implement when needing any form of respite. This clinician also stated that when time in nature is spent with others, it generates conversation. Additionally, clinicians reflected on the negative effect that lack of contact with nature would have on their stress levels and feeling overwhelmed. One clinician described the need to schedule more walks in, as a “good balm to both sadness, anger, frustration, general stress, that it just brings all of those emotions down and…promotes happiness” (07), especially when life was stressful. Stepping into nature was described as stepping outside of the human world, and clinicians valued this use of their time. They also reflected on the sensory experiences that come with nature and these include the calming, relaxing, and peaceful aspects of the sights, smells, and sounds. All these personal experiences allowed clinicians to realise the therapeutic aspects of time spent in nature.

#### Recommending and/or facilitating engagement with nature to mental health consumers

Many clinicians encouraged consumer participation in nature-based activities such as going for walks, with some comparing it to taking compulsory medication. Spending time in nature was described as pivotal for achieving holistic health outcomes and adding meaning to consumers’ lives. As one clinician simply stated, “No matter what else happens, [nature] will still be there in one way, shape, or form” (03). As clinicians were prompted to comment on their view of NBIs, some highlighted the convenience of engaging in therapy outside of the normal office setting. They felt that nature pervades the local landscape and access to it can be generally inexpensive. One clinician stated:

…because of the area we live in, engaging with nature is quite easy. So, whether it’s going to the beach or going to the escarpment…walking around the lake…it’s fairly easy to access something close by, which then also means that it’s low cost (12).

Some clinicians also believed that NBIs would enhance rapport and therapeutic alliance: “it's a way to build better rapport and better relationships that that are more natural than this forced clinical relationship” (09). A few clinicians described that they already incorporate nature into their clinical work. For example, one clinician moved the therapy session to a natural environment perceiving it to have beneficial outcomes:

I had a very difficult client, and it was really complex, so much trauma, and I took him to the botanic gardens. The first thing we did was just to feed the ducks. Then we walked around and looked around, and then it became our meeting place (…). And then from then on, I could see the improvement (13).

If NBIs such as nature walking groups were to be organised within their services, clinicians expressed willingness to discuss and promote these programs with their consumers as a valid treatment option. Clinicians also described NBI groups as potentially appealing for mental health consumers because the responsibility of navigating the walk lies on the facilitator.

### Perceived benefits of nature-based interventions for mental health consumers

Clinicians perceived numerous potential benefits of NBIs for mental health consumers such as relaxation, alleviated mental health symptoms, social connections, confidence and empowerment, and physical exercise resulting in better health.

#### Mindfulness and relaxation

Providers believed NBIs would provide space for mindfulness, relaxation, calmness, and restorative sensory experiences. One clinician elaborated on these potential benefits in the following way:

…being aware of what’s going on in the environment around me, whether it be that something is flowering or whether it be that a bird flies over. Almost like a calming meditative [experience] and I think people would benefit from that calming meditative feel about it. You feel part of something. You feel part of the environment. You feel part of the world and I think people might find that really useful. And just a lot of awareness of outside yourself (02).

Nature was thus viewed as allowing consumers to step outside their own headspace and create a sense of connectedness to something bigger outside oneself. Clinicians furthered explored the benefits of the sensory experiences that could result from NBIs. Many described tactile sensations such as: “walking on grass with bare feet” (02), “bellbirds whistling in the trees” (04), “the smell of the sea breeze” (12), and “the earth in my fingers” (04). These experiences were believed to calm and relax, soothe feelings of sadness, anger, and frustration, and stimulate a sense of resilience and happiness. Facilitators’ skilled guidance of NBIs such as leading nature mindfulness exercises could further strengthen these benefits.

#### Alleviated mental health symptoms

Clinicians believed NBIs would offer several short- and long-term benefits for management of mental health issues especially anxiety and depression. For example, nature walks would release endorphins and increase feelings of pleasure and enjoyment which can improve a person’s mood. One clinician described it in the following way:

People were able to enjoy this walk, and you could see the difference in people [...] every day in the morning, they were able to move away from that environment into nature. And when these people came back, they were totally different. You could see smiles in their face (14).

Similarly, some clinicians perceived that NBIs would provide a space for consumers to “have a break from their internal mayhem and briefly focus on things outside of themselves…” (05). At the same time, as one clinician explained: “…it also helps them to be able to identify and put words to how they feel, and how they express things, and connect that with what they’re thinking but also what their body is feeling” (09).

#### Social connections

Clinicians believed that NBIs would create opportunities for social connections and camaraderie, which were described as vital for mental health. NBIs offered in groups, such as nature walking groups, could help break down loneliness and social withdrawal tendencies which were identified as common among mental health consumers. As one clinician stated: “it might be the only social interaction they get at all” (03). Clinicians emphasised that consumers should not be expected to socialise and talk to other group members if they chose not to. They hoped, however, that by being in the presence of others, consumers would slowly acquaint themselves and create some connections with group members. For example, while walking, consumers could engage in informal conversations without the potentially intimidating eye-contact and intensity of a therapy room. As one clinician explained, walking with others “generates a flow of conversation different to that when you are in a sit-down space” (11). Moreover, they discussed the potential for consumers to meet like-minded people, to motivate each other to take ownership of their recovery and to gain perspective from others who have similar lived experiences and are not closely linked to their personal lives. Group NBIs would thus emphasise the idea that consumers are not alone on their journey and normalise mental health issues in general.

#### Alternative intervention

Numerous clinicians have advocated for NBIs as an alternative to the medical model that would entice consumers who struggled with regular sit-down therapy, taking medications, and leaving the house. A few clinicians specifically described the potential of NBIs for teenagers given their tendencies to rebel against traditional interventions and the expectation to be processing and expressing their emotions in the formal and structured environment of a therapy room. Similarly, one clinician explained: “…I think when they feel like someone’s not staring at them from across a room expecting a certain answer, when you’re just going for a walk, it kind of breaks down that clinical barrier…” (09). NBIs thus alleviate the described feeling of being locked in a room for a therapy session and create “an environment that’s more conducive to mental health support” (09). Group NBIs can also create a familiar, safe, and facilitated opportunities for leaving home regularly, which may be of particular benefit for consumers afraid to go out alone.

#### Confidence building and empowerment

Clinicians considered how NBIs such as nature walking groups could support consumers’ functioning and enhance their sense of empowerment and confidence in the recovery. As consumers make the decision to actively look after themselves, they start to recognise their power and ability to make changes in their lives and be successful in achieving their treatment goals. Referring to an existing NBI within a mental health program, one clinician summarized, “Our staff would lead a group walk every morning for consumers. So, the consumers’ comments are that they just feel re-energised. They feel like they have developed some sort of confidence about themselves” (14). Clinicians also believed that NBIs could enhance consumers’ confidence and self-esteem as they discover that spending time in nature can help with self-regulation and stress management.

#### Physical exercise

Some clinicians discussed physical health benefits as many NBIs promote exercise: “improving physical health, walking, it's high on our agenda” (13). Clinicians identified that mental health consumers tend to have poorer physical health and thus, any form of physical activity was believed to benefit consumers. Exercise would additionally support management of medication side-effects such as weight gain or lethargy.

### Perceived barriers to nature-based interventions within mental health services

Despite a having a strong belief in, and being supportive of NBIs, clinicians expressed concerns about the development, facilitation, and participation in nature-based activities such as walking groups. They listed several individual and organisational barriers to the implementation of NBIs including consumers’ resistance and mental health symptoms, limited access, and safety risk.

#### Individual barriers

Clinicians believed that mental health consumers may experience 1) resistance, scepticism, and a lack of awareness of the NBI’s benefits; 2) poor physical health and not feeling fit; 3) mental health symptoms (e.g., lack of motivation, anxiety); 4) fear of having to socialize and/or not fitting into the group; and 5) access issues (e.g., lack of transportation), all of which may prevent them from participating in NBIs.

Clinicians perceived that some consumers might not be willing to participate in NBIs due to scepticism and unawareness of its benefits. Clinicians highlighted that some people are oblivious to the natural environment and thus dismissive of its health and wellbeing benefits. One clinician expected consumers may undermine the value of nature walks in advance: “Oh, what’s the point of this, to go on a walk in the middle of nowhere?” (08). Some clinicians believed that their consumers would need to be convinced into participation, and that consumers would not necessarily engage or believe in the benefits even if they did attend. One clinician described her consumer’s response to a recommendation to spend more time in nature: “…the idea of going for a bushwalk just seemed quite weird to her like, ‘Why? Why would you do that? What are we going to do?’ she said” (05). Consumers’ resistance to join NBIs, according to clinicians, may develop around their motivation or physical limitations: “Lack of motivation, lack of energy, lack of belief in the process, just don't like walking. A lot of people just don't like exercise. What else would they stop them? Physical injuries” (01). Clinicians thus believed that consumers who did not enjoy exercise and/or were not physically fit would be particularly reluctant to join.

Mental health symptoms and their management were also raised as potential barriers to NBIs. For example, clinicians worried that consumers with anxiety and social phobia might feel uneasy about being in public spaces and concerned about a potential panic attack in front of others. A clinician described the resistance of a consumer to NBIs in the following way: “the reason she doesn’t want to do it is because she’s socially anxious and she’s worried about people watching her while she’s walking and what they think of her” (07). Clinicians also spoke about the potential for consumers to be preoccupied with the symptoms they experience, and how potential sensory experiences need to be considered, for example: “being a low-intensity kind of sensory environment would be a good match for people with psychosis” (12). Lastly, some clinicians added that special consideration is required for consumers who may have suicidal ideations: “Clearly, if you’ve got a suicidal consumer, you’re not going to walk beside a cliff. You might not want to go into too remote an area with them. You don’t want to get stuck in the bush…” (03).

If NBIs such as nature walking groups were to be offered, clinicians believed the group makeup and dynamics may pose challenges to some consumers. For example, younger consumers might not want to join a group with older adults due to physical abilities and levels of fitness which can impact the group’s pace and because socially it would not be considered a “cool thing to do” (11). Clinicians also discussed the presentation and severity of mental illnesses:

Some people that we work with can be quite unwell and others can present as being fairly functioning and fairly well. So also, sometimes, people, young people within that age group, the person who’s sort of presenting well is going to be like, “I’m not unwell like that person, I’m not coming” (06).

Due to existing mental health stigma, some consumers could feel ashamed to walk with the group in a community setting. Finally, some might feel intimidated by a group setting or become withdrawn due to the inability to socialise with others.

Finally, geographic accessibility was perceived as a barrier to NBIs. Many mental health consumers do not drive and/or do not have access to a car. Some clinicians believed that consumers would find accessing NBIs via public transport inconvenient further decreasing their motivation to participate. A few clinicians also mentioned that accessing NBIs may be especially difficult for consumers with limited financial resources and thus providing transport would be essential.

#### Organisational barriers

Clinicians also discussed barriers from the perspective of the mental health services and organisations. They listed things such as: “risk assessment” (03), “having the managers see the value in it” (07), and “limited understanding or effort to understand the effectiveness” (11). All clinicians believed that mental health services prioritize the medical treatment model, which is viewed as evidence-based and taking place in a controlled environment. This opened the discussion around the organisation’s doubt and uncertainty of the evidence of NBIs for mental health. As one clinician identified:

I think the service doing anything new is always a barrier because they always want to know, “Is there an evidence base?” and where we’re taking the time from? Like what are the staff not doing instead of doing this group? They don’t want to do anything that doesn’t save them time somewhere else, to make up for the time lost (07).

Many clinicians believed that, like the consumers, the health system is unaware and/or sceptical about the positive effects of NBIs. Clinicians described that within their organisations certain staff members may be unwilling to change or implement new ideas, especially when the intervention is to take place in an uncontrolled environment, such a park or bush. Clinicians worried that running NBIs which would take away time from their current prescribed responsibilities. Overall, the main difficulty was having to justify the value of NBIs, proving that it is a legitimate form of treatment rather than an unnecessary cost that poses risks.

Finally, safety risk was frequently discussed as organisations would require a risk assessment and management to implement NBIs as part of their services. Potential risks included the inability to complete a long walk; consumers becoming unwell; managing behavioural issues; consumer vulnerability and sensitivity; and accidents (e.g., falls). Some clinicians believed that organisational risk management strategies can be so extreme that they may completely block NBIs. As one clinician explained: “If you want to do anything that is outside of the office, it’s just fraught with red tape and barriers, and having to write risk policies and ‘risk this’, and ‘risk that’, because the environment is not controlled” (09). Clinicians emphasised that given the risk averse culture community mental health services, these potential issues would create a variety of obstacles to implementing NBIs such as a nature walking group.

## Discussion

This study explored the perceptions of mental health clinicians on the benefits of, and barriers to implementing NBIs within community mental health services. The results indicate that clinicians were supportive of, and were willing to, recommend NBIs to their consumers. Clinicians perceived that participation in NBIs would enhance consumers’ mental wellbeing, social connections, mindfulness, and relaxation beyond the outcomes obtained from current mental healthcare provision. Nevertheless, the clinicians listed several barriers including consumer resistance, scepticism, and unawareness of the potential benefits, as well as organisational factors that might inhibit implementation of NBIs in mental health settings. Importantly, the findings demonstrated that clinicians participating in the study spent time in nature and actively promoted exposure to nature to their consumers. Clinicians appreciated and utilised nature-based activities in their daily routines such as nature walks to alleviate stress and to find time for reflection. They believed nature had a healing effect. Their willingness to recommend NBIs to consumers appeared to be linked with their personal connection with nature.

This study’s findings illustrate that participating clinicians unanimously agreed that NBIs would contribute positively to mental health consumers’ recovery journeys. Consistent with the Attention Restoration Theory [54] and the Stress Recovery Theory [55], clinicians believed that nature would have a stress-reducing and restorative effect on the consumers. As discussed by other authors [5, 13, 27], clinicians perceived NBIs as an innovative treatment for mental illness that would offer new ways of managing and alleviating symptoms, especially for depression and anxiety. As traditional mental health treatments do not suit all consumers, NBIs were seen as a more appealing, alternative, and non-medical form of intervention. This is consistent with Glover’s [7] recovery-focused approach which challenges the institutionalised responses to mental illnesses and calls for interventions that take a person-centred and holistic view of mental health and wellbeing. Clinicians implied that NBIs would encourage consumers to explore new ways of managing mental health symptoms, building their confidence in the ability to recover. Finally, consistent with the existing evidence that links NBIs with social connections [11, 12], clinicians emphasized that NBIs would alleviate consumers’ loneliness and isolation. The ability to just ‘be’ in the presence of others would benefit consumers, regardless of if they chose to directly interact with group members.

Nevertheless, clinicians anticipated a variety of barriers to implementing NBIs as part of mental health services, only some which have been discussed in the literature [12]. When considering these potential barriers, it is important to remember that they are based on clinicians’ assumptions, and the perceptions and experiences of mental health consumers are yet to be explored. Clinicians listed consumers’ resistance, scepticism, and unawareness of nature’s benefits as potential barriers to participation. Although Robertson et al. [44] acknowledged the difficulty in motivating mental health consumers to engage in nature, clinicians claimed that lack of motivation when depressed or the severity of mental illness would be a major hinderance to participation. Thus, adjustments to the NBIs, such as viewing nature scenes instead of a bushwalk, may be required for consumers experiencing the acute phase of mental illness or those with suicidal ideations, until their active symptoms subside. Additionally, clinicians worried that factors such as age, levels of physical fitness, or limited social skills and social anxiety could impact the group dynamics. The presence of others while engaging in NBIs may thus be a benefit and barrier for different people. Similar to some of the organisational barriers to NBIs listed by Shanahan et al. [12], clinicians believed that mental health services may be resistant to NBIs due to the reliance on traditional treatment approaches and perceptions of safety risk and potential liability issues for the organisation if a consumer was to suffer an injury. It is possible, however, that barriers to implementation of NBIs at the organisational level may be, to a large extent, due to lack of knowledge about NBIs and difficulty in changing behaviours of health professionals [12].

### Strengths and limitations

There are various strengths of this study. The in-depth exploration clinicians’ perceptions of NBIs is essential, given that the success of NBIs within mental health services depends, to a large extent, on clinicians’ buy-in and support. The study participants included social workers, psychologists, peer workers, and mental health nurses, which is important given the multidisciplinary nature of mental health services. Finally, questions about the impact of nature on the clinicians’ personal lives were included, which permitted exploration of clinicians’ motivations and potential investment in these interventions.

The study was conducted in one local health district, known for rich biodiversity and easy access to nature, which may be viewed as a limitation to the universality of the study. Although mental health services based in large cities may not have access to the same natural environments, green spaces in urban areas such as parks, community gardens (e.g., therapeutic horticulture), or botanical gardens can be utilised for NBIs [17, 32]. Additionally, the study was limited to mental health clinicians only. A follow-up study is currently being planned with mental health consumers to explore their perspectives and interest in NBIs. Finally, it is likely that the clinicians who volunteered to be interviewed were attracted to the research because of their personal interest and connection with nature, which may create some bias in the findings. Despite these limitations, we believe the study has some implications for mental health services and future research.

### Implications for mental health practice and research

Our findings suggest that the ability to implement NBIs involving mental health clinicians in the Australian context may depend on organisations’ ability to reframe treatment strategies both conceptually (e.g., shift towards recovery-oriented, holistic models of care) and operationally (e.g., staff workload allocations to facilitate these groups). The potential scepticism and lack of awareness of nature’s benefits on mental health needs to be addressed in both the mental health consumers and clinicians; especially those who have the power to decide whether NBIs should be considered. This could involve professional development opportunities such as NBI training and conferences for staff, and education workshops and materials to build consumers’ knowledge about benefits of nature exposure on mental health. Participation in co-produced randomised trials will also increase organisational knowledge, support, and trust in NBIs. Additionally, consumers should actively participate and collaborate with clinicians in co-designing, organising, and facilitating NBI sessions. Consumer involvement early on and throughout these processes should ensure that consumers’ needs and preferences are being met and encourage participation [25].

Given the limitations of this study and the gaps in the existing literature, there is ample room for future studies. Future research should strive to establish the effectiveness and sustaining benefits of NBIs within mental health services. For example, clinical trials should be carried out to compare the outcomes of consumers participating in NBIs with those in “treatment as usual” groups. Moreover, studies need to explore mental health consumers’ perceptions of and experiences with NBIs which can then inform the development of future NBIs. Research is also required to determine the knowledge and perceptions of mental health service managers and policymakers on the benefits of and barriers to NBIs as their support is required to implement these interventions. Lastly, future research should compare the interest and capacity of mental health organisations located in both urban and regional areas to understand how NBIs need to be adjusted based on the environmental context.

## Conclusion

There is an increasing need for the health systems around the world to consider alternative interventions to alleviate mental health symptoms. Nature-based interventions may be such an alternative and as this study demonstrated mental health clinicians were supportive of implementing NBIs as part of mental health services. Many clinicians strongly believed in the restorative qualities of nature and were indeed passionate about both spending time themselves in nature and connecting their consumers with natural environments. Building on this passion and the existing evidence of the positive impact of nature on human wellbeing, indicate the potential for routine implementation of NBIs within mental health services and ultimately improving the treatment outcomes of service recipients.

## Data Availability

The datasets used and/or analysed during the current study are available from the corresponding author on reasonable request.

## Abbreviations

NBIs: Nature-based interventions
ISLHD: Illawarra Shoalhaven Local Health District
NSW: New South Wales
